# Alcoholic and non-alcoholic steatohepatitis: global perspective and emerging science

**DOI:** 10.1007/s00535-018-01542-w

**Published:** 2019-01-14

**Authors:** Gurmehr Brar, Hidekazu Tsukamoto

**Affiliations:** 1 Southern California Research Center for ALPD and Cirrhosis, Department of Pathology, Keck School of Medicine, University of Southern California, 1333 San Pablo Street, MMR-402, Los Angeles, CA 90033 USA; 2 Greater Los Angeles VA Healthcare System, Los Angeles, CA USA

**Keywords:** Alcoholic steatohepatitis, Non-alcoholic steatohepatitis, Non-alcoholic fatty liver disease, High-fat diet, Risk factors

## Abstract

Alcohol and high-fat diet are two major risk factors responsible for metabolic diseases, which are manifested as steatohepatitis and liver cancer in the liver, and chronic pancreatitis and pancreatic adenocarcinoma (PDAC) in the pancreas. These metabolic diseases are becoming increasingly prevalent around the globe, and more importantly, their two major etiologies commonly coexist to precipitate the disease processes. To highlight the importance of these metabolic diseases, Japanese Society of Gastroenterology (JSGE) and National Institute on Alcoholism and Alcohol Abuse of National Institute of Health cosponsored the JSGE’s 7th International Forum jointly held with the 12th International Symposium on ALPD and Cirrhosis. Toward the main theme of “Frontiers in ASH, NASH, NBNC-HCC and PDAC”, this platform showcased presentations by 12 invited international and Japanese speakers on brain–gut–liver interactions, emerging mechanisms of ASH and NASH, metabolic reprogramming, and new therapeutic targets for cirrhosis, HCC, and PDAC. This editorial discusses the most recent data on global statistics on how alcohol and obesity impact health and longevity as a prelude to a brief summary of the symposium presentations and discussions, primarily focusing on the first two session themes.

## Alcohol abuse and obesity as leading global risk factors for death and disability

**Fig. 1:**
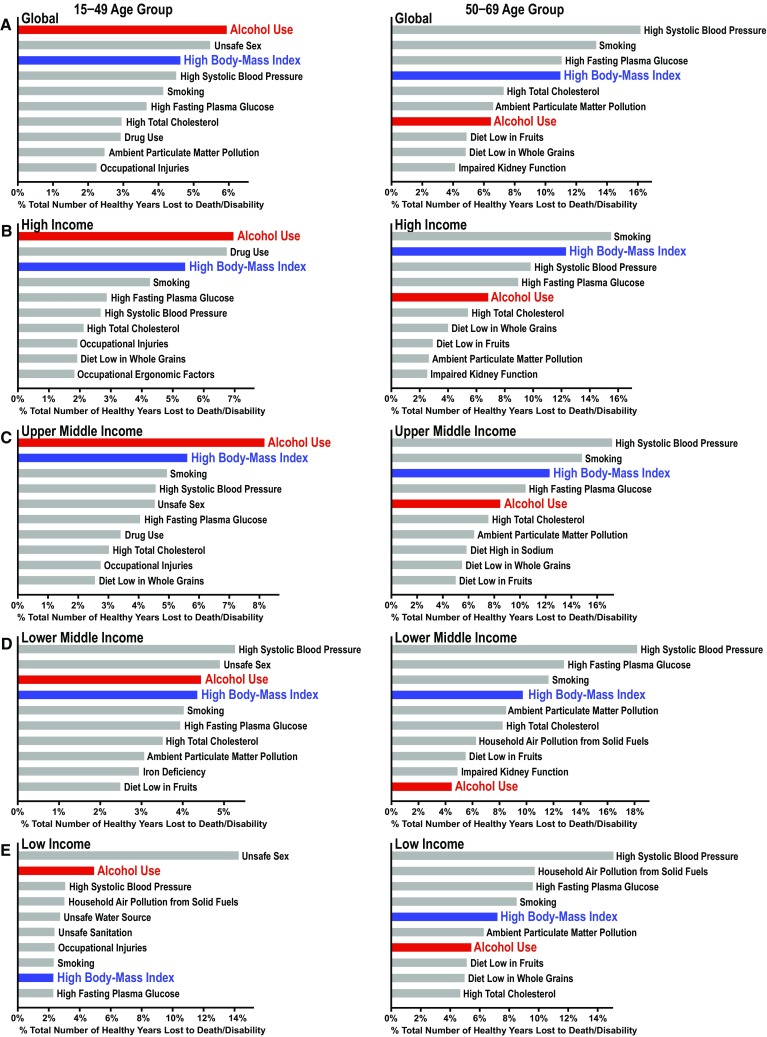
Top ten risk factors as determined by the percentage of lost healthy years (disability-adjusted life years) per risk factor among the 15–49 age group (left) and 50–69 age group (right) globally (**a**), in high-income countries (**b**), in upper middle-income countries (**c**), in lower middle-income countries (**d**), and in low-income countries (**e**). Note that alcohol is the top leading risk factor for death and disability worldwide and among high-and upper middle-income nations. High body mass index (BMI) is the third risk factor globally and is ranked at 2nd–4th among lower-income to high-income nations at the 15–49 age group, and similar trends are also seen in the 50–69 age group

Alcohol abuse and high body mass index (BMI) are pressing health issues that globally and significantly contribute to burdens of disease not only in nations of high and middle economic standing but also in nations of poverty. The strongest evidence for this conclusion is found in the Global Burden of Disease 2016 report published by the Institute for Health Metrics and Evaluation at the University of Washington [1, 2]. The data from this report have been modified to depict ten leading risk factors that contribute to the burdens of disease among an age-group population of 15–49 years (Fig. 1, left) vs. 50–69 years (Fig. 1, right) globally (Fig. 1a), in high-income countries (Fig. 1b), upper middle-income countries (Fig. 1c), lower middle-income countries (Fig. 1d), and low-income countries (Fig. 1e). This income-based grouping of countries was based on their classification by the World Bank using gross national income (GNI) per capita (low-income economies are those with a GNI per capita of $995 or less in 2017; lower middle-income economies are those with a GNI per capita between $996 and $3895; upper middle-income economies are those with a GNI per capita between $3896 and $12,055; high-income economies are those with a GNI per capita of $12,056 or more) [3]. We selected the age group of 15–49 years as it generally represents the most productive population, allowing the assessment of the risk factors’ effects on the overall economy and growth of a nation. The risk factors are listed with their corresponding effects as the percentage of disability-adjusted life years (equivalent to the percentage loss of healthy life years). According to the data, alcohol use is the most significant risk factor for death and disability globally (Fig. 1a) and in both high-income and upper middle-income countries (Fig. 1b, c), responsible for an approximately 5.9% reduction in healthy life years globally and a higher 7–8% reduction in middle to affluent nations. These nations include developed countries such as the United States and Canada, as well as countries that are currently undergoing a surge of economic development such as Thailand and Malaysia. In low-income nations, alcohol use is second to a dominant leading risk factor of unsafe sex with a 5% loss of healthy life years. These findings indicate that alcohol use is a leading global risk factor throughout a whole spectrum of the economic standing, and alcohol use may further compound the ability of low-income nations to prosper which are in many cases, already plagued by the prevalence of infectious disease and high child mortality. As the world becomes increasingly globalized in terms of communication, economy and lifestyle, it is essential that modifiable risk factors rooted in emerging social and cultural trends, such as alcohol use, be closely examined by the international community.

Similarly, globalization has had a significant impact on the roles that high-fat diets and soft drinks high in fructose play in determining the death and disability worldwide. These diets and drinks contribute to increased BMI, raising the risk for a host of metabolic diseases including non-alcoholic fatty liver disease (NAFLD). While high BMI as a risk factor was once limited to developed countries, its effect is now evident throughout a larger fraction of the world. High BMI is ranked as the third risk factor globally with an overall 4.6% loss of healthy years, and ranked 3rd and 2nd in high and upper middle-income nations claiming 5.4% and 5.6% reductions, respectively. In low-income countries, this percentage drops to 2.3%, indicating that the extent of “Westernization” of lifestyle appears to predicate this epidemiologic difference. Perhaps of greatest concern is that the percentages in middle- and low-income countries have increased from 4.9% and 1.6% in 2010, respectively, indicating that the number of healthy life years lost due to a high BMI is increasing even in non-affluent nations.

## NAFLD/NASH pathology in non-obese patients

Non-alcoholic steatohepatitis (NASH), a severe form of NAFLD is a significant cause of chronic liver disease worldwide. Overtime, the presence of risk factors, such as weight gain, diabetes, hypertension, menopause, and genetic conditions, promotes the development of NASH, liver fibrosis, cirrhosis, and hepatocellular carcinoma (HCC) [4]. Obesity is considered a primary cause of NAFLD and a majority of patients with NAFLD are either overweight or obese. For this reason, obese individuals are routinely screened for fatty liver disease. However, NAFLD has been reported in lean and non-obese individuals, particularly in Asian populations. Due to their non-obese status, these individuals may not be screened for NAFLD, as clinical guidelines advise screening for patients who are overweight or diabetic [5], placing them at risk for the progression to NASH and HCC. Moreover, NAFLD in non-obese populations demonstrates that BMI may not be the sole primary risk factor for the development of the disease, and other factors are involved in such patients. Lean NAFLD patients generally present with less severe symptoms than obese patients. In a recent study from the United States, using data from the National Health and Nutrition Examination Survey III (NHANES III), the prevalence of NAFLD in lean individuals was 7.4% and these patients appeared less likely to develop characteristic metabolic symptoms such as insulin resistance, diabetes mellitus, hypercholesteremia, and hypertension [6]. This trend is further supported by population studies in Korea [7], Japan [8], China [9], and India [10]. Further, the metabolic characteristics of lean NAFLD vary across ethnicities. For instance, lean, non-alcoholic, non-diabetic, non-smoking Asian Indians have at least a twofold higher risk of developing insulin resistance and fatty liver than matched Caucasians, Hispanics, Blacks and Eastern Asians [11]. Identifying underlying mechanisms for such ethnic differences is essential for the development of ethnically personalized medicine for NAFLD.

## Moderate alcohol use as a risk factor for NAFLD/NASH?

**Fig. 2:**
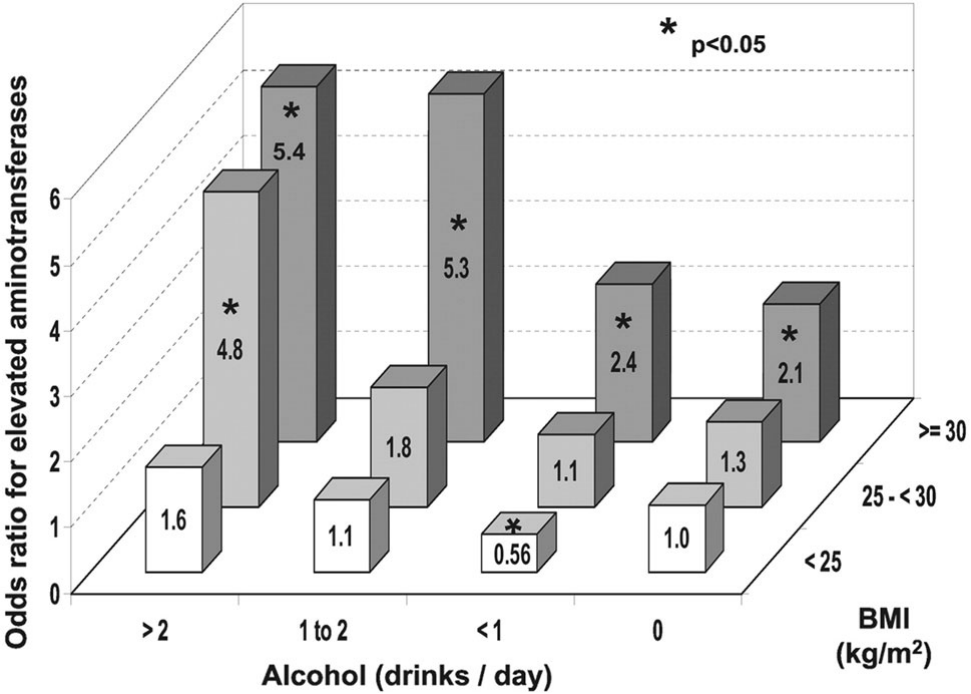
Multivariate-adjusted logistic regression ORs for elevated serum aminotransferase activity as function of alcohol intake and BMI. Asterisk indicates p < 0.05 compared with normal weight nondrinkers. Note mutually positive-interactive effects by alcohol intake and BMI. (Reprinted with permission from [13])

Alcohol and obesity are two independent risk factors for loss of healthy life years and for the development of ASH and NASH for our interest. But interactions among the two are also important. For instance, chronic, excessive alcohol use, as determined by daily intake of > 40 g/kg for men and > 20 g/kg for women, synergistically promotes liver disease in NAFLD patients, particularly in overweight or obese subjects [12–14]. As a result, NAFLD patients are generally advised to minimize or avoid alcohol consumption [15]. However, NAFLD patients are diagnosed based on all the required diagnostic criteria plus alcohol intake of less than the threshold alcohol dose (30 g/kg for men and 20 g/kg for women), and how moderate alcohol intake below these threshold levels influences liver disease in these patients is an outstanding question. Existing literature reveals conflicting results on this question regarding NAFLD and NASH and in many cases, limited by the methodological factors such as failure to assess lifetime alcohol use, the pattern of alcohol intake, and the effects of confounding factors [16]. For instance, many studies supporting the notion that moderate alcohol use has a protective effect on NAFLD progression are cross-sectional or retrospective, limiting the ability to assess temporal associations and accurate self-reported alcohol use [17–21]. These studies also largely failed to assess the patterns of alcohol use, such as heavy episodic drinking, limiting the full characterization of “moderate alcohol use” as determined by averaged daily intake [15]. In a large cross-sectional study involving 16,573 individuals examining the effects of alcohol intake and BMI on plasma aminotransferases, a multivariate-adjusted logistic regression analysis revealed a significantly reduced odd ratio (OR = 0.59) for elevated plasma aminotransferases by < 1 drink/day among those with < 25 BMI but in contrast, a significantly increased risk (OR = 2.4) by the same moderate <1 drink/day among those with > 30 BMI, underscoring the important influence of obesity on the effect of moderate alcohol intake as shown in Fig. 2 [13]. Although the data cannot be extrapolated for the implication in liver disease, this analysis indeed revealed mutually positive-interactive relationship by the two risk factors: synergistically increased OR by moderate alcohol intake in obese but not lean individuals (BMI > 30) and conversely by moderate increased BMI (25–30) in heavy but not moderate drinkers (> 2 drinks/day). A population-based study in Germany found that being overweight or obese significantly increased the progression of liver disease when coupled with moderate alcohol use [22, 23]. There is even some evidence that moderate alcohol use may promote tumor incidence in NASH patients. A retrospective study on patients with NASH cirrhosis in the US showed significantly increased incidence of HCC by moderate alcohol intake [24].

As alcohol use and increased BMI are prevalent and constitute two major risk factors around the world, there is an urgent need to assess cross-interactions of the two by a large prospective multi-ethnic cohort study with accurate assessment of the amount, frequency, and pattern of alcohol intake along with careful qualitative and quantitative measurements of other lifestyle factors such as diet and exercise. Genetic influence on these interactions is also of obvious interest. Such a study should better delineate environmental and genetic factors which determine the predisposition to harmful cross-interactive effects of alcohol and obesity.

## Emerging science in metabolic dysregulation, ASH and NASH

In light of the global importance of alcohol and obesity-associated metabolic diseases, the 7th JSGE International Forum jointly held with the 12th International Symposium on ALPD and Cirrhosis was a timely and enlightening event for the JSGE community. It showcased 12 invited speakers with the main theme on frontiers in metabolic liver and pancreatic diseases. The space allocated for this editorial is not sufficient to cover all symposium presentations in depth and thus it will review and summarize the essence of the presentations by 5 US speakers on the two selected topics of brain–gut–liver interactions and emerging mechanisms of NASH and ASH.

## Brain–gut–liver interactions

Organ cross talk is a communal and essential mechanism of acute and chronic diseases, particularly metabolic liver diseases. The importance of intestinal microbiota in maintaining the homeostasis of essentially all organs in the body is now well recognized. It is not surprising that excessive or/and unbalanced nutrients or alcohol intake, the primary etiologies of NAFLD and ASH disturb gut flora as a most upstream event. Dysbiosis (microbial imbalance) alters microbial metabolites (e.g., short chain fatty acids, lactate, tryptophan and tyrosine metabolites) which normally support and protect intestinal epithelium; reduces production of anti-microbial peptides; and enhances paracellular and transcellular translocation of bacteria and pathogen-associated molecular patterns (PAMPs). This stimulates an innate immune response in the intestine and the liver, and early pathogenetic changes in NAFLD and ASH. Proinflammatory activation of hepatic macrophages and hepatic stellate cells initiates PAMP-driven inflammatory and fibrogenic responses in the liver, which are coupled to organelle stress (mitochondrial, ER, lysosomal) and activation of cell death pathways in hepatocytes to initiate ASH and NASH. These changes are mirrored in the brain as microglia and astrocyte proinflammatory activation and neurodegeneration via cross talk, yet to be elucidated but being actively investigated.

Both NAFLD and ALD are dictated by metabolic alternations which are closely linked to altered peripheral circadian clock regulation due to the abnormal nutrient or alcohol intake as well as dysbiosis as *Professor Paolo Sassone*-*Corsi of University of California Irvine* discussed in his opening lecture. This metabolic reprogramming is in part mediated by histone and non-histone acetylation regulated by the NAD-dependent sirtuins (SIRT1, 3, and 6). NAD+ level exhibits circadian rhythmicity because the rate-limiting enzyme for the NAD salvage pathway, nicotinamide mononucleotide phosphoribosyltransferase (NAMPT), is transcriptionally upregulated by the circadian clock core transcription factors, CLOCK:BMAL [25]. As such, NAD+ generated by CLOCK-regulated NAMPT activates SIRT1, deacetylates and activates acetyl-CoA synthetase1 (AceCS1), which generates acetyl-CoA for protein acetylation [25]. High-fat diet impairs CLOCK:BMAL chromatin recruitment [26] and thus inhibits this NAMPT–NAD–SIRT1–AceCs1 pathway affecting many metabolic pathways, including lipid metabolism [26]. High-fat diet causes gut dysbiosis which raises the amplitude of oscillating PPARγ target genes in the liver via epigenetic and transcriptional activation, leading to the development of fatty liver, and these events are abrogated by gut sterilization by antibiotic treatment [27]. As gut dysbiosis and a reduced NAD/NADH ratio also occur after alcohol consumption, these regulations are also likely relevant to ALD.

*Bernd Schnabl of University of California, San Diego* enlightened the audience on new developments in the understanding of the pathogenetic implications that gut dysbiosis renders for ALD, particularly via altered gut microbial metabolites. Such examples, discovered by a combined application of metagenomic sequencing and metabolomic analysis, include upregulation of bacterial cholylglycine hydrolase which deconjugates bile acids (BA) and reduces intestinal levels of unconjugated BA, leading to suppressed FXR-mediated expression of FGF-15 by ileum, derepression of *Cyp7a1*, and increased BA synthesis in alcohol-fed mice. Administration of the intestine-restricted FXR agonist or overexpression of FGF-19 (a human FGF-15 ortholog), modulated hepatic genes involved in lipid metabolism and ameliorated ASH, suggesting a causality of dysbiosis-mediated suppression of the BA–FXR–FGF15 signaling [28]. He also discussed the importance of intestinal fungi in ALD by demonstrating the ability of the fungal PAMP β-glucan to activate hepatic macrophages and IL-β release and damaging hepatocytes in alcohol-fed mice and intestinal Candida overgrowth in alcoholic patients was discussed as the clinical relevance [29].

*Gyongyi Szabo of University of Massachusetts Medical Center* followed to summarize the pathway of liver inflammation in ASH involving TLR-PAMP-mediated priming and NLRP3 canonical inflammasome-induced IL-β activation triggered by DAMPs such as uric acid [30]. She extended this mechanism to neuroinflammation in alcohol-fed mice through demonstration of upregulation of acetylated and phosphorylated HMGB and the HMGB1 receptors (TLR2, TLR4, TLR9, and RAGE), NLRP3/ASC-dependent caspase 1 and IL-1β activation, and induction of TNF-α and MCP-1 in the brain of alcohol-fed mice [31]. Further, this neuroinflammation was associated with upregulation of miR155 and prevented by miR155 deficiency, suggesting the role of this miRNA [32]. These discussions highlighted the converging action of alcohol on liver and brain inflammatory signaling.

## Emerging mechanisms for ASH and NASH

*Arun Sanyal of Virginia Commonwealth University* opened this session by discussing novel treatments of NASH. He emphasized the importance of defining and standardizing the clinical endpoints pursued by clinical trials [33]. One such endpoint for NASH early stage 1–3 is to reduce the disease activity and at least prevent the progression of fibrosis to cirrhosis, and more desirably, regress fibrosis. Toward this goal, the insulin sensitizer, PPAR-γ agonist pioglitazone was shown to reduce steatosis, inflammation and ballooning of hepatocytes with some evidence of reduction of fibrosis [34, 35]. However, it has side effects of weight gain, fluid retention, and osteopenia. A recent trial with the PPAR-α/δ agonist elafibranor demonstrated an improvement of cardiometabolic parameters but equivocal effects on liver parameters. Reanalysis of the data based on NASH with high disease activity and using revised guidelines appeared to significantly improve liver histology. FXR agonists have also received much attention. Obeticholic acid improved all parameters of early stage of NASH but was associated with increased LDL-cholesterol. New small molecule FXR agonists may improve NASH without raising LDL-cholesterol. A potential mechanism of the FXR agonist’s therapeutic effect may be mediated by the release of FGF-19 by ileum. A recombinant FGF-19 analog which does not have growth promoting and pro-tumorigenic effects has been shown to reduce hepatic fat and liver enzymes in biopsy-proven NASH patients. Glucagon-like peptide (GLP)-1 is an intestinal hormone which stimulates insulin secretion, decreases liver glucose production, increases satiety by delaying gastric emptying, and provides cardioprotective effects. Liraglutide, a long-acting GLP-1 agonist improved NASH histology and key metabolic parameters such as weight, BMI, and LDL-cholesterol levels in a small pilot study. Studies with semaglutide, a GLP-1 agonist requiring only weekly administration, are currently under way. The CCR2-CCR5 antagonist, cenicriviroc, which blocks chemotactic signaling of CCL2 (MCP-1), CCL3 and 4 (MIP-1α and β), and CCL5 (RANTES), showed encouraging phase 2b study results of improving fibrosis without worsening NASH activity and a phase 3 study is currently under way. Another drug which advanced to phase 3 trial is selonsertib, a ASK-1 inhibitor which showed significant histologic improvement of liver fibrosis without worsening NASH activity. Other drugs are also actively being tested, as summarized in the excellent review articles [34, 35].

*Vijay Shah of Mayo Clinic* discussed therapeutic opportunities for ASH by taking an intriguing approach of exploiting similarities and differences with NASH. ASH and NASH have overlapping histologic and clinical features which are likely caused by common underlying mechanisms. These common mechanisms include gut dysbiosis, hepatocellular organelle stress, inhibited autophagy, altered hepatic lipid metabolism, innate immune activation, and hepatic stellate cell activation [36]. In addition, from the early discussions of this symposium and recent literature, disrupted peripheral clock and extracellular vesicles (EVs) may be added. As for differences, signaling pathway immediately downstream of TLR4 requires MyD88 for NASH but appears to be MyD88 independent for ASH. Insulin resistance and its metabolic consequences drive NASH, but not as much for ASH although insulin resistance may be caused by binge drinking [37]. NLRP3-inflammasome and IL-1β activation is causal in early ASH [30] but its involvement in NASH is yet to be determined. Although EVs released from fatty hepatocytes have proinflammatory and pro-fibrogenic effects, their cargos carry different miRNAs and proteins. They may serve as diagnostic markers as well as experimental tools to identify unique mechanisms of intercellular or inter-organ cross talk in the pathogenesis of NASH vs. ASH.

These two sessions were followed by the session on Metabolic Reprogramming in Liver and Pancreatic Diseases, encompassing the presentations on lipid reprogramming linking liver fibrosis to cancer by *Hidekazu Tsukamoto of University of Southern California*; cross talk between mitochondria and autophagy in pancreatitis by *Anna Gukovskaya of University of California, Los Angeles*; and the role of the vagus nerve in hepatic glucose metabolism and NAFLD progression by *Hiroshi Inoue of Kanazawa University*. The final session on New Therapeutic Targets for Cirrhosis, HCC, and PDAC included the presentations on neutrophils, the NF-kB p50 subunit and exercise as interventional targets in liver inflammation and cancer by *Derek Mann of Newcastle University*; Metavert, a new therapeutic for treatment and prevention of metastasis in PDAC by *Stephen Pandol of Cedars*-*Sinai Medical Center*; precision medicine for HCC and pancreatic cancer by *Shuji Tanaka of Tokyo Medical and Dental University*; and epigenetics in liver fibrosis by *Jelena Mann of Newcastle University*.

The international symposium on alcoholic liver and pancreatic diseases (ALPD) and cirrhosis began in 2006 with a charge by National Institute on Alcoholism and Alcohol Abuse (NIAAA) of US National Institute of Health to pursue a well-defined mission of promoting global exchange of research knowledge and international collaborations concerning ALPD and cirrhosis plaguing the world. Global statistics are staggering: the WHO reports more than 3 million deaths due to alcohol misuse, corresponding to nearly 6% of all deaths in the world. As summarized in the introduction of this editorial, alcohol use and high BMI, which cause ASH and NASH, respectively, are major risk factors for disease and death around the globe, reducing our healthy life span by 5–7%. The symposium aims to face this challenge of the global epidemic by promoting international exchange of cutting-edge basic and translational sciences as witnessed in the 12th symposium in Tokyo. We would like to thank all the speakers who contributed their science to the symposium; the JSGE and Professor Kazuhiko Koike for sponsoring and providing a symposium platform as part of the 104th JSGE General Meeting; and NIAAA for its conference Grant (R13AA020697) enabling the continuation of the symposium.
